# Estimated annual direct medical costs of manifestations among patients with activated phosphoinositide 3-kinase delta syndrome

**DOI:** 10.1007/s10238-025-01773-1

**Published:** 2025-07-12

**Authors:** Nicholas L. Hartog, Eveline Y. Wu, Nicholas L. Rider, Yang Meng, Brian Hartline, Philippe Adams, Saurabh Aggarwal, Amanda Harrington

**Affiliations:** 1 Corewell Health, Grand Rapids, MI USA; 2 Michigan State University College of Human Medicine, Grand Rapids, MI USA; 3 Division of Pediatric Allergy/Immunology and Rheumatology, Department of Pediatrics, University of North Carolina, Chapel Hill, NC USA; 4 Department of Health Systems & Implementation Science, Virginia Tech Carilion School of Medicine, Section of Allergy-Immunology, The Carilion Clinic, Roanoke, VA USA; 5 Lumanity, Morristown, NJ USA; 6 Pharming Healthcare, Inc., Warren, NJ USA; 7 NOVEL Health Strategies, Chevy Chase, MD USA

**Keywords:** Activated phosphoinositide 3-kinase delta syndrome, APDS, Estimated direct medical costs, Manifestations, Annual prevalence of manifestations in patients with APDS, Economic burden

## Abstract

Activated phosphoinositide 3-kinase delta syndrome (APDS) is a rare genetic disease associated with heterogeneous manifestations, including recurrent infections, lymphoproliferation, and autoimmunity. This analysis sought to estimate the mean annual direct medical costs of manifestations associated with APDS. A burden-of-illness cost calculator was developed based on survey responses from the US clinical experts, published evidence, and the US cost sources (2023). Results from the survey provided estimates of mean annual prevalence of various manifestations associated with APDS and the mean number of times patients experienced recurrent manifestations in a year. In the base case analysis, the annual mean cost of manifestations associated with APDS per patient in the US was estimated to be $116,387 (range, $10,711–$417,455) with gastrointestinal-, infection-, and hematology-related manifestations being the largest contributing factors. The weighted average scenario analysis resulted in similar estimates of mean annual manifestation costs as the base case analysis. To our knowledge, our study is the first to provide estimates of annual prevalence of manifestations associated with APDS and to estimate the annual direct medical costs for patients with APDS in the US. The high mean annual cost associated with APDS contributes to the economic burden of patients and health care payers. Patients with APDS often require off-label, symptomatic treatments for various manifestations that do not target the root cause of the disorder. These treatments are associated with high costs that may not control symptoms. This analysis provides valuable support for discussions about resource utilization and the economic burden of APDS.

**Supplementary Information:**

The online version contains supplementary material available at 10.1007/s10238-025-01773-1.

## Introduction

Activated phosphoinositide 3-kinase delta syndrome (APDS) is a rare inborn error of immunity [1, 2]. First characterized in 2013, APDS is associated with significant, persistent, and progressive clinical consequences [1–3]. Gain-of-function variants in the *PIK3CD* gene or loss-of-function variants in the *PIK3R1* gene encoding phosphoinositide 3-kinase (PI3K) delta result in increased activation of the PI3K/AKT/mammalian target of rapamycin (mTOR)/S6K signaling pathways in immune cells, which leads to immunodeficiency and immune dysregulation [1–6].

APDS is rare, with an estimated prevalence of 1–2 per million people [1]. Patients with APDS present with a variety of clinical manifestations, which are often chronic and progressive in nature, and may require off-label, symptomatic treatments that do not target the root cause of the disorder. Common manifestations include inflammatory or autoimmune disease, malignancy, increased risk of infections, including recurrent respiratory tract infections, and neurodevelopmental delay and growth retardation [3, 4]. The estimated median overall survival is 64 years in patients with APDS [7]. Lymphoma and infections have been reported as the cause of death in 24% and 22% of patients with APDS, respectively [8].

Although management of manifestations in patients with APDS can include multiple therapies and surgical interventions, the specific medical costs associated with APDS remain unclear due to limited data and variability in treatment approaches [3, 4]. Given the recent characterization of APDS in 2013, and with only 351 unique patients with APDS reported globally in the literature as of 2023, the economic burden of manifestations associated with APDS has not been well established [8]. The objective of this study was to estimate the mean annual direct medical costs of manifestations associated with APDS in the US.

## Methods

### Cost calculator

**Fig. 1:**
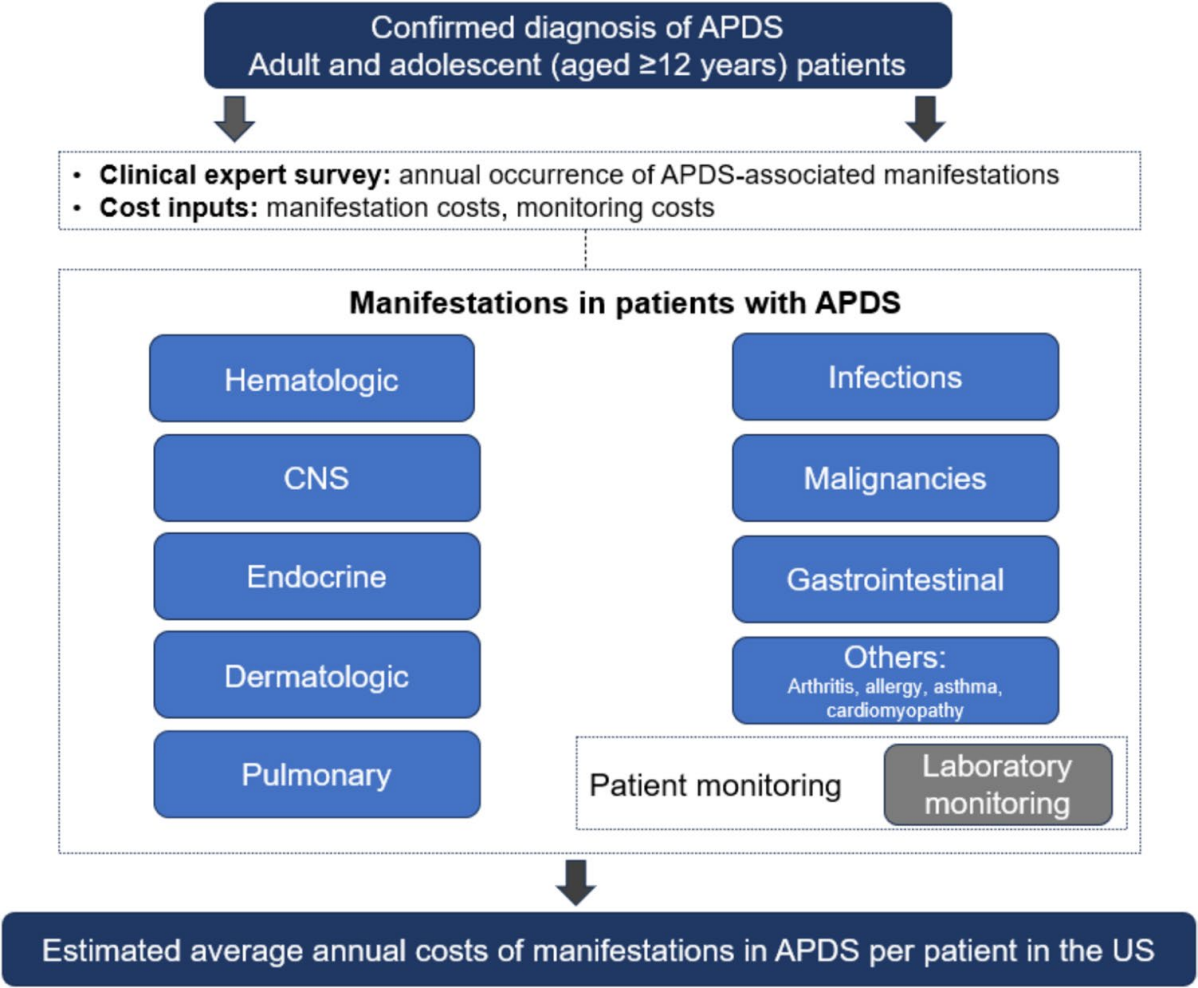
Cost model. *APDS* activated phosphoinositide 3-kinase delta syndrome and *CNS* central nervous system

To estimate the mean annual cost incurred by the health care system for a patient with APDS in the US, we developed a burden-of-illness cost model reflecting the perspective of US commercial payers (Fig. 1). The model combined clinical expert survey data on the annual prevalence of APDS manifestations with the US costs data related to manifestations associated with APDS. The inputs included the results of a clinical expert survey on manifestations and the cost data. Only the US direct medical costs were included in the model. The patient population comprised adults and adolescents 12 years and older with a diagnosis of APDS. Patient characteristics in the model, such as weight and body surface area, were sourced from a randomized, placebo-controlled, phase 3 trial in APDS [9]. The model did not account for patient subgroups and only considered a 1-year time horizon. Given that the model calculates annual manifestation costs, it was not necessary to discount the model results.

#### Clinical expert survey

After review of the scientific literature and discussion with clinicians, a list of manifestations associated with APDS was compiled [10–13]. Annual rates of manifestations were not available in the published literature to inform the model, given the rarity of the disease. A comprehensive survey was conducted to gather information relating to the annual prevalence of manifestations associated with APDS as well as the frequency at which they were experienced in clinical practice each year.

The survey content was developed from data from APDS-specific literature [10–13]. For each question, the annual prevalence was defined as the percentage of patients who were likely to have experienced a manifestation each year. Survey respondents were asked to assume that the manifestations were being treated and to consider all patients with APDS, not just those who they have directly treated. For example, those who did not have firsthand experience with the manifestations identified by the publications were asked to consider the experiences of APDS experts with whom they interact to estimate prevalence. This request of clinicians was required due to the rareness of the disease.

Six US-based clinicians who had experience treating patients with APDS responded to the survey. Collectively, survey respondents had treated a total of 27 patients, with a range of 2–8 patients per respondent at the time of the survey. With only 351 unique patients described globally in the literature as of 2023 and considering the rarity of APDS, the patient ranges reported among respondents are meaningful and highlight their expertise in APDS [8]. Two means were calculated from the clinician survey responses: (1) a standard arithmetic mean and (2) a weighted mean. For the weighted mean, weights were based on the number of patients treated by the clinician; greater weight was assigned to survey responses from clinicians who treated more patients with APDS.

#### Cost estimates

##### Manifestation costs

Approximately 150 manifestation costs were used as inputs in the model and were categorized as infection (e.g., bacilli Calmette-Guérin disease, cellulitis, chronic rhinosinusitis, dental/oral tissue abscesses, endocarditis, fungal infection, granulomatous lesions, meningitis, ocular infections, otitis media, otitis needing tympanostomy tubes, pneumonia, respiratory tract infections, septicemia, Epstein–Barr virus, cytomegalovirus, herpes simplex virus, varicella-zoster virus, tonsillitis, and tonsillitis with tonsillectomy), hematology (e.g., cytopenias, pancytopenia, factor XI deficiency, factor IX deficiency, hemolytic anemia, hepatomegaly, immune thrombocytopenia, lymphadenopathy, nodular lymphoid hyperplasia, spherocytosis, splenomegaly, and thrombotic thrombocytopenic purpura), malignancy (e.g., diffuse large B-cell lymphoma [DLBCL], Hodgkin lymphoma, mucosa-associated lymphoid tissue [MALT] lymphoma, marginal zone B-cell lymphoma, multiple lymphoma, and other non-lymphoma malignancies), central nervous system (e.g., neuropsychiatric disorders, developmental delays, failure to thrive, seizures, and brain biopsy), pulmonary (e.g., atelectasis, bronchiectasis, and interstitial lung disease), gastrointestinal (e.g., enteropathy, graft-vs-host disease, eosinophilic esophagitis/eosinophilic gastrointestinal disease, inflammatory bowel disease, nodular regenerative hyperplasia of the liver, pancreatic insufficiency, gastrointestinal surgery, liver biopsy, and liver transplant), dermatology (e.g., eczema and dermatitis), endocrine (e.g., adrenal insufficiency, diabetes, and hypothyroidism), and other (e.g., rheumatoid arthritis, allergy, asthma, and cardiomyopathy). Manifestation costs were sourced from the literature.

##### Patient monitoring costs

Patient monitoring costs related to disease surveillance and testing costs incurred by patients were sourced from Centers for Medicare & Medicaid Services (CMS). Supplementary Table 1 presents the cost per test, and the total cost per year as well as the testing frequencies assumed in the model. Some costs, such as chemistry panel laboratory test and computed tomography scans, were derived from the sum and the mean of multiple CMS costs codes, respectively (Supplementary Table 2). CMS costs and other Medicare costs were inflated to commercial prices using a factor of 134%, as per the Medicare Payment Advisory Commission (MedPAC) source [14].

##### Factors affecting manifestation treatment costs

All cost inputs were inflated using the Consumer Price Index to represent 2023 prices (US dollar) (Supplementary Table 3). The frequency of manifestations associated with APDS was based on the results of the clinician survey, using standard means of survey responses in the base case and weighted means in the scenario analysis. For manifestations that can recur within a year (e.g., pneumonia), clinicians were asked to report the number of times in a year a patient may experience the manifestation; the reported number of episodes was included in the cost calculation for that manifestation. The inputs derived from the manifestations survey were included in the calculations for the proportion of patients who experienced manifestations associated with APDS and the resource use associated with each manifestation.

An example sequence of the cost calculation for pneumonia is provided in Supplementary Fig. 1.

### Statistical analysis

A base case analysis applying the standard mean and a scenarios analysis using weighted mean results from the survey (referred to hereafter as weighted mean scenario analysis) were performed. In the base case analysis, values derived from the survey results were used to calculate the standard arithmetic mean, while the minimum and maximum results were used to determine the lower and upper estimates, respectively. The output from each analysis was the annual cost of manifestations associated with APDS per patient.

### Ethics approval

This article is based on clinical expert survey data and publicly available cost information and does not contain any new studies involving human participants or animals conducted by any of the authors.

## Results

### Manifestation survey

Responses from the clinician survey were grouped by category of manifestation: infections, hematologic, malignancies, pulmonary, gastrointestinal, endocrine, central nervous system, and other (Table 1). The infections with the highest estimated mean annual prevalence were respiratory infections (85%), chronic sinusitis/rhinosinusitis (47%), pneumonia (37%), and otitis media (33%). Of the six survey respondents, five clinicians reported annual prevalence rate ranges of 25%–90% and 10%–75% for chronic sinusitis/rhinosinusitis and otitis media, respectively; one clinician reported rates of 0% for both manifestations. The hematologic manifestations with the highest estimated mean annual prevalence were lymphadenopathy (71%), splenomegaly (67%), and hepatomegaly (34%). Malignancies reported in the survey included various lymphomas, with the highest prevalence rates occurring with DLBCL (3%) and Hodgkin lymphoma (3%). Of the six survey respondents, one clinician reported an annual prevalence rate of 0% for DLBCL and Hodgkin lymphoma, and two clinicians reported 0% for multiple lymphoma, MALT lymphoma, and marginal zone B-cell lymphoma. Ranges reported by the other clinicians were 1%–5% for DLBCL, Hodgkin lymphoma, and multiple lymphoma and 1%–2% for MALT lymphoma and marginal zone B-cell lymphoma. The estimated mean annual prevalence of autoimmune diseases was 48%.

**Table 1:** Estimated annual prevalence of manifestations associated with APDS and estimated number of annual episodes of recurrent manifestations

Manifestation	Mean annual prevalence, %	Minimum annual prevalence, %	Maximum annual prevalence, %
*Infections*			
Respiratory infections	85	80	90
Chronic sinusitis/rhinosinusitis	47	0^a^	90
Pneumonia	37	15	67
Otitis media	33	0^a^	75
Severe or persistent herpesvirus infection	20	0^a^	50
Fungal infections	6	0^a^	10
Septicemia	6	0^a^	20
*Hematologic*			
Lymphadenopathy	71	50	100
Splenomegaly	67	25	100
Hepatomegaly	34	20	50
Cytopenia	31	0^a^	50
Nodular lymphoid hyperplasia	17	0^a^	50
Immune thrombocytopenia	14	0^a^	30
Hemolytic anemia	13	0^a^	25
*Malignancies*			
Diffuse large B-cell lymphoma	3	0^a^	5
Hodgkin lymphoma	3	0^a^	5
Multiple lymphoma	2	0^b^	5
MALT lymphoma	1	0^b^	2
Marginal zone B-cell lymphoma	1	0^b^	2
*Pulmonary*			
Bronchiectasis	40	10	90
Atelectasis	16	0^a^	50
Interstitial lung disease	13	3	25
*Gastrointestinal*			
Enteropathy	24	20	30
Diarrhea	22	0^a^	50
Inflammatory bowel disease	8	0^a^	20
*Endocrine*			
Hypothyroidism	11	0^a^	25
Diabetes	10	2	25
Adrenal insufficiency	4	0^a^	10
*CNS*			
Failure to thrive	10	0^a^	20
Seizures	5	0^b^	10
*Other*			
Asthma	37	5	100
Dermatitis/eczema	18	0^a^	50
Cardiomyopathy	6	0^a^	25

Recurrent manifestations	Mean annual episodes per patient, No.	Minimum annual episodes per patient, No.	Maximum annual episodes per patient, No.
Otitis media	2.00	0.00^c^	3.00
Fungal infections	1.50	0.00^c^	5.00
Chronic sinusitis/rhinosinusitis	1.33	0.00^c^	3.00
Severe or persistent herpesvirus	1.33	0.00^c^	2.00
Pneumonia	1.17	1.00	2.00

^a^Of the six survey respondents, one clinician reported an annual prevalence rate of 0%

^b^Of the six survey respondents, two clinicians reported an annual prevalence rate of 0%

^c^Of the six survey respondents, one clinician reported 0 as the mean number of annual episodes. The same clinician also reported an annual prevalence rate of 0% for these manifestations

*APDS* activated phosphoinositide 3-kinase delta syndrome, *CNS* central nervous system, and *MALT* mucosa-associated lymphoid tissue

Clinicians also provided estimates of the mean number of annual episodes per patient of recurrent manifestations (Table 1). The recurrent manifestations with the highest estimated mean number of annual episodes per patient were otitis media with a mean of 2.00 episodes per patient and fungal infections with 1.50 episodes per patient. A mean of 1.33 episodes per patient annually was estimated for chronic sinusitis/rhinosinusitis and severe or persistent herpesvirus infection, and 1.17 episodes per patient annually for pneumonia. Of the six survey respondents, one clinician reported 0 as the mean number of annual episodes per patient for otitis media, fungal infections, chronic sinusitis/rhinosinusitis, and severe or persistent herpesvirus infection. The same clinician also reported an annual prevalence rate of 0% for these manifestations. Ranges reported by the other clinicians were 1.00–3.00 for otitis media, 1.00–5.00 for fungal infections, 1.00–3.00 for chronic sinusitis/rhinosinusitis, and 1.00–2.00 for severe or persistent herpesvirus infection.

### Cost model

Results from the base case analysis estimated the mean annual cost of manifestations associated with APDS per patient at $116,387, with a range of $10,711–$417,455 (Table 2). The highest costs were associated with manifestations related to the gastrointestinal tract ($32,573), infections ($31,711), and hematology ($20,452), contributing substantially to the overall mean annual cost per patient (Table 2). Malignancies, endocrine, dermatology, and central nervous system manifestations were the smallest contributing factors to the mean annual cost of manifestations associated with APDS per patient (Table 2).

**Table 2:** Base case analysis results of mean annual costs of APDS per patient; costs account for manifestations incurred annually across the heterogeneous APDS population

Manifestation	Mean annual costs per patient	Minimum annual costs per patient	Maximum annual costs per patient
Gastrointestinal	$32,573	$202	$99,698
Infections	$31,711	$6720	$126,968
Hematologic	$20,452	$460	$108,531
Pulmonary	$10,311	$2160	$23,848
Others	$9251	$945	$26,350
Malignancies	$7571	$0	$16,458
Endocrine	$2583	$223	$6239
Dermatologic	$1036	$0	$5482
CNS	$899	$0	$3882
*Total annual cost of manifestations associated with APDS per patient*^*a*^	*$116,387*	*$10,711*	*$417,455*

^a^Sums of costs in each column may not equal total costs because of rounding

*APDS* activated phosphoinositide 3-kinase delta syndrome and *CNS* central nervous system

**Fig. 2:**
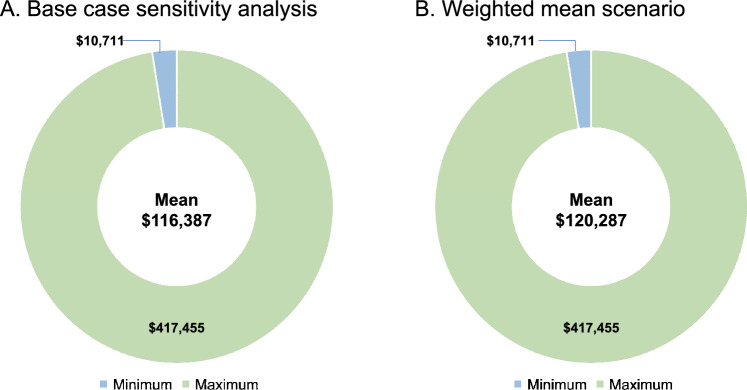
Total (min, max) annual costs of manifestations associated with APDS per patient by **A** Base case analysis and **B** Weighted mean scenario. *APDS* activated phosphoinositide 3-kinase delta syndrome

The weighted mean scenario analysis estimated the mean annual costs of manifestations associated with APDS per patient at $120,287 showing consistent results when compared with the base case analysis (Fig. 2).

## Discussion

Patients with APDS can experience a wide range of manifestations requiring various treatments, which can lead to high health care costs [3, 10, 11, 13]. Health care system resource use and allocation for patients with APDS are limited by the general sparsity of literature specific to health care costs associated with APDS. To our knowledge, this is the first study to estimate the annual direct medical costs related to manifestations associated with APDS in the US.

Results from our clinician survey provide, to our knowledge, the first annual rates of manifestations reported for patients with APDS. The previous reports were retrospective reviews and provided results more akin to lifetime rates of manifestations [2, 10, 11, 15]. Therefore, comparing our estimated annual mean prevalence rates with previously published rates is difficult. The timeframes differ, and the characterization of several manifestations varies among the previous reports when compared with our survey results. However, in alignment with the previous reports, our results show that respiratory-related infections as well as lymphoproliferative disease, including lymphadenopathy and splenomegaly, are common in patients with APDS [2, 10, 11, 15]. Overall, our results further illustrate the heterogeneity of APDS-associated manifestations.

The base case analysis showed that individual mean annual health care cost of manifestations in patients with APDS was $116,387, with the potential to increase to $417,455 in patient cases consisting of more severe combinations of APDS manifestations. Manifestations contributing the most to costs were gastrointestinal, infections, and hematology. The consistent results of the base case analysis and the weighted mean scenario analysis give the model results a higher degree of internal validity, given that these methods of analyses were able to account for various types of uncertainty in the model inputs. Although various types of lymphomas had estimated mean annual prevalence rates between 1% and 3%, the estimated annual prevalence rate of lymphoma was up to 19% in our study. Hodgkin lymphoma and DLBCL were estimated as the most common types of lymphoma, which aligns with the previous reports [11, 15]. These findings are clinically relevant, as the primary observed cause of death in patients with APDS has been reported to be lymphoma [8].

In our study, infections were one of the main contributors of mean annual costs of manifestations associated with APDS per patient, which was not surprising based on the lifetime prevalence of infections reported in the literature [10, 11, 15]. In a retrospective study of 53 patients with APDS, recurrent respiratory and recurrent otitis media infections were reported in 98% and 49% of patients, respectively, with chronic rhinosinusitis reported in 45% of patients [10]. Severe or persistent herpesvirus infections were reported in 49% of patients [10]. Similar findings were observed in a retrospective study of 36 patients with APDS2, in which upper respiratory tract (otitis media and sinusitis) and lower respiratory tract infections were observed in 100% and 77% of patients, respectively [15]. In a systematic review of 253 patients with APDS, the most common infectious manifestations were respiratory tract infections including pneumonia (43.6%), otitis (28.8%), and sinusitis (25.9%) [11]. Infections in patients with APDS are a serious concern and have been reported as the cause of death in 9 of 41 patients with APDS (22%), second only to lymphoma, which was the observed cause of death in 10 of 41 patients with APDS (24%) [8]. Therefore, infections may influence the median overall survival in patients with APDS, which is estimated to be 11 years shorter relative to the global population [7]. Additionally, patients with APDS and concurrent lymphoma have up to 23.5% lower median overall survival compared to those without lymphoma [7].

### Strengths and limitations

The key strength of this model is the focus on results from expert clinician surveys reporting the prevalence of manifestations. The surveys represent the views of clinicians who treat APDS, and given the rarity of the condition, the number of patients represented provides valuable insight into the burden of manifestations and treatments. However, the survey data are limited; the survey relied on the ability of clinician respondents to recall information accurately and the assumption that their experience with patients with APDS was representative of the natural history of the disease. The model integrates these clinician insights directly into its calculations, contributing to its validity in a therapeutic area characterized by limited and inconsistent evidence. Additionally, the model incorporates data from published literature and standardized cost sources relevant to a US commercial payer perspective.

APDS is a rare disease that was not characterized until 2013, and there is limited research specific to patients with APDS and virtually no published accounts of the costs of manifestations associated with APDS [1]. All of these factors serve as a major limitation of modeling costs of APDS-associated manifestations and subsequent treatments. Cost inputs are derived from a variety of the US-based publications not directly related to APDS. Therefore, the results are estimates based on proxy cost sources and lack external validation of actual costs incurred in real-world clinical practice.

## Conclusions

To our knowledge, this is the first study to estimate the annual direct medical costs for manifestations associated with APDS in the US. Patients with APDS can experience a range of manifestations requiring various treatments, which can lead to high costs [10, 11]. The findings of this study suggest that manifestations associated with APDS are heterogeneous, with many estimated to occur at an annual prevalence rate of over 50%. These manifestations can cost up to an estimated $417,455 per patient each year, thereby underscoring the need for effective and safe treatment for patients with APDS. Future work in this area could focus on real-world evidence specific to APDS-related costs and increase evaluation and comprehension of the cost-effectiveness of various treatments. An *International Classification of Diseases, Tenth Revision* (*ICD-10*) code for APDS became effective in 2022 and can now be used to evaluate health care resource use and costs in other data sources. Due to the lack of existing information on this disease state, this analysis is valuable to support discussions about resource utilization and economic implications in APDS.

## Supplementary Information

Below is the link to the electronic supplementary material.

Supplementary file1 (DOCX 161 KB)

## Data Availability

Data are provided within the manuscript or supplementary materials.

## References

[1] Vanselow S, Wahn V, Schuetz C. Activated PI3Kδ syndrome—reviewing challenges in diagnosis and treatment. Front Immunol. 2023;14:1208567. 37600808 10.3389/fimmu.2023.1208567 PMC10432830

[2] Maccari ME, Wolkewitz M, Schwab C, Lorenzini T, Leiding JW, Aladjdi N, et al. Activated phosphoinositide 3-kinase δ syndrome: update from the ESID Registry and comparison with other autoimmune-lymphoproliferative inborn errors of immunity. J Allergy Clin Immunol. 2023;152(4):984-96.e10. 37390899 10.1016/j.jaci.2023.06.015

[3] Coulter TI, Cant AJ. The treatment of activated PI3Kδ syndrome. Front Immunol. 2018;9:2043. 30245694 10.3389/fimmu.2018.02043 PMC6137162

[4] Michalovich D, Nejentsev S. Activated PI3 kinase delta syndrome: from genetics to therapy. Front Immunol. 2018;9:369. 29535736 10.3389/fimmu.2018.00369 PMC5835040

[5] Cant AJ, Chandra A, Munro E, Rao VK, Lucas CL. PI3Kδ pathway dysregulation and unique features of its inhibition by leniolisib in activated PI3Kδ syndrome and beyond. J Allergy Clin Immunol Pract. 2024;12(1):69–78. 37777067 10.1016/j.jaip.2023.09.016 PMC10872751

[6] Durandy A, Kracker S. Increased activation of PI3 kinase-δ predisposes to B-cell lymphoma. Blood. 2020;135(9):638–43. 31942637 10.1182/blood.2019002072

[7] Mahendran M, Upton JEM, Ramasubramanian R, Memmott HL, Germain G, Büsch K, et al. Overall survival among patients with activated phosphoinositide 3-kinase delta syndrome (APDS). Orphanet J Rare Dis. 2025;20(1):212. 40319290 10.1186/s13023-025-03734-z PMC12049806

[8] Büsch K, Memmott HL, McLaughlin HM, Upton JEM, Harrington A. Genetic etiologies and outcomes in malignancy and mortality in activated phosphoinositide 3-kinase delta syndrome: a systematic review. Adv Ther. 2025;42(2):752–71. 39636570 10.1007/s12325-024-03066-7 PMC11787279

[9] Rao VK, Webster S, Šedivá A, Plebani A, Schuetz C, Shcherbina A, et al. A randomized, placebo-controlled phase 3 trial of the PI3Kδ inhibitor leniolisib for activated PI3Kδ syndrome. Blood. 2023;141(9):971–83. 36399712 10.1182/blood.2022018546 PMC10163280

[10] Coulter TI, Chandra A, Bacon CM, Babar J, Curtis J, Screaton N, et al. Clinical spectrum and features of activated phosphoinositide 3-kinase δ syndrome: a large patient cohort study. J Allergy Clin Immunol. 2017;139(2):597-606.e4. 27555459 10.1016/j.jaci.2016.06.021 PMC5292996

[11] Jamee M, Moniri S, Zaki-Dizaji M, Olbrich P, Yazdani R, Jadidi-Niaragh F, et al. Clinical, immunological, and genetic features in patients with activated PI3Kδ syndrome (APDS): a systematic review. Clin Rev Allergy Immunol. 2020;59(3):323–33. 31111319 10.1007/s12016-019-08738-9

[12] Maccari ME, Abolhassani H, Aghamohammadi A, Aiuti A, Aleinikova O, Bangs C, et al. Disease evolution and response to rapamycin in activated phosphoinositide 3-kinase δ syndrome: the European society for immunodeficiencies-activated phosphoinositide 3-Kinase δ syndrome registry. Front Immunol. 2018;9:543. 29599784 10.3389/fimmu.2018.00543 PMC5863269

[13] Oh J, Garabedian E, Fuleihan R, Cunningham-Rundles C. Clinical manifestations and outcomes of activated phosphoinositide 3-kinase δ syndrome from the USIDNET cohort. J Allergy Clin Immunol Pract. 2021;9(11):4095–102. 34352450 10.1016/j.jaip.2021.07.044 PMC8578310

[14] MedPAC. Medicare payment advisory commission—medicare payment policy. In: Executive summary. 2023. Available at: https://www.medpac.gov/wp-content/uploads/2023/03/Mar23_MedPAC_Report_To_Congress_SEC.pdf.

[15] Elkaim E, Neven B, Bruneau J, Mitsui-Sekinaka K, Stanislas A, Heurtier L, et al. Clinical and immunologic phenotype associated with activated phosphoinositide 3-kinase δ syndrome 2: a cohort study. J Allergy Clin Immunol. 2016;138(1):210-8.e9. 27221134 10.1016/j.jaci.2016.03.022

