# Supplementary material for: Estimated annual direct medical costs of manifestations among patients with activated phosphoinositide 3-kinase delta syndrome
Source: Clin Exp Med. 2025 Jul 12;25(1):246. doi: 10.1007/s10238-025-01773-1 (PMC12255542; doi:10.1007/s10238-025-01773-1)
Supplement: Supplementary file 1 — Supplementary file1 (DOCX 161 KB) [file 10238_2025_1773_MOESM1_ESM.docx]

**Supplementary Information**

**Estimated Annual Direct Medical Costs of Manifestations Among Patients With Activated Phosphoinositide 3-Kinase Delta Syndrome**

***Clinical and Experimental Medicine***

Nicholas L. Hartog, MD^1,2^; Eveline Y. Wu, MD^3^; Nicholas L. Rider, DO^4^; Yang Meng, PhD^5^; Brian Hartline, MD^6^; Philippe Adams, MBA^6^; Saurabh Aggarwal, PhD^7^; Amanda Harrington, PhD^6^

^1^Corewell Health, Grand Rapids, MI, USA; ^2^Michigan State University College of Human Medicine, Grand Rapids, MI, USA; ^3^Department of Pediatrics, UNC School of Medicine, Chapel Hill, NC, USA; ^4^Virginia Tech Carilion School of Medicine, Roanoke, VA, USA; ^5^Lumanity, Bethesda, MD, USA; ^6^Pharming Healthcare, Inc., Warren, NJ, USA; ^7^NOVEL Health Strategies, Chevy Chase, MD, USA

Corresponding author: Amanda Harrington, PhD

A.Harrington@pharming.com

**Supplementary Table 1** Patient-monitoring cost inputs

**Supplementary Table 2** Chemistry panel laboratory test and CT scan cost inputs

**Supplementary Table 3** APDS manifestation costs inputs

**Supplemental Fig. 1** Methodology for calculating manifestation costs in patients with APDS

**Supplemental Table 1:** Patient-monitoring cost inputs^1,2^

| Monitoring cost | Cost per test (2023 USD – commercial costs) | Frequency per year | Cost per year (2023 USD – commercial cost) | Source |
| --- | --- | --- | --- | --- |
| Clinical assessment immunologist | $296.07 | 4 | $1,184.29 | CMS PFS |
| CBC with differential laboratory test | $10.41 | 6 | $62.47 | CMS LFS |
| Immunoglobulin panel laboratory test | $37.39 | 3.33 | $124.62 | CMS LFS |
| Chemistry panel laboratory test | $103.25 | 5.67 | $585.07 | CMS LFS |
| B-cell flow cytometry | $80.53 | 2.67 | $214.73 | CMS PFS |
| T-cell flow cytometry | $133.05 | 2.67 | $354.80 | CMS PFS |
| CT scan, PET scan, or MRI for patients with APDS (any/all areas) | $313.95 | 1 | $313.95 | CMS PFS |
| Clinical assessment, pulmonologist | $151.21 | 4 | $604.82 | CMS PFS |
| Clinical assessment, infectious disease specialist | $151.21 | 4 | $604.82 | CMS PFS |
| Clinical assessment, gastroenterologist | $151.21 | 4 | $604.82 | CMS PFS |
| Spirometry FEV testing | $36.33 | 1.33 | $48.44 | CMS PFS |
| EBV PCR monitoring | $57.41 | 1.33 | $76.54 | CMS LFS |

*APDS* activated phosphoinositide 3-kinase delta syndrome, *CBC* complete blood cell count, *CMS* Centers for Medicare & Medicaid Services, *CT* computed tomography, *EBV* Epstein-Barr virus, *FEV* forced expiratory volume, *LFS* laboratory fee schedule, *MRI* magnetic resonance imaging, *PCR* polymerase chain reaction, *PET* positron emission tomography, *PFS* physician fee schedule, *USD* US dollar

**Supplemental Table 2:** Chemistry panel laboratory test and CT scan cost inputs^1,2^

| **CMS code** | **Short description** | **Cost (2023 USD Medicare costs)** |
| --- | --- | --- |
| **Chemistry panel laboratory costs** | | |
| 80053 | Metabolic panel | $10.56 |
| 82040 | Albumin | $4.95 |
| 82247 | Bilirubin, total | $5.02 |
| 82310 | Calcium | $5.16 |
| 82374 | CO_2_ (bicarbonate) | $4.88 |
| 82435 | Chloride | $4.60 |
| 82565 | Creatinine | $5.12 |
| 82947 | Glucose | $3.93 |
| 84075 | Alkaline phosphatase | $5.18 |
| 84132 | Potassium | $4.76 |
| 84155 | Protein | $3.67 |
| 84295 | Sodium | $4.81 |
| 84460 | ALT | $5.30 |
| 84450 | AST | $5.18 |
| 84520 | Urea nitrogen | $3.93 |
| Chemistry panel laboratory test total (Medicare costs) |  | $77.05 |
| Chemistry panel laboratory test total (converted to commercial costs) |  | $103.25 |
| **CT scan average costs derived from multiple CMS codes** | | |
| 70470 | CT head/brain without/with contrast | $182.99 |
| 70482 | CT orbit/ear/fossa without/with contrast | $222.30 |
| 70488 | CT maxillofacial without/with contrast | $194.85 |
| 72127 | CT neck spine without/with contrast | $208.07 |
| 72130 | CT chest spine without/with contrast | $210.10 |
| 70492 | CT soft tissue neck without/with contrast | $233.82 |
| 71270 | CT thorax diagnostic without/with contrast | $207.05 |
| 72133 | CT lumbar spine without/with contrast | $208.75 |
| 73202 | CT upper extremity without/with contrast | $263.98 |
| 73702 | CT lower extremity without/with contrast | $205.70 |
| 72194 | CT pelvis without/with contrast | $268.73 |
| 74170 | CT abdomen without/with contrast | $278.21 |
| 74178 | CT abdomen and pelvis without contrast followed by contrast in 1 or both body regions | $361.24 |
| CT scan average cost (Medicare costs) | | $234.29 |
| CT scan average cost (converted to commercial costs) | | $313.95 |

*ALT* alanine aminotransferase, *AST* aspartate aminotransferase, *CMS* Centers for Medicare & Medicaid Services, *CT* computed tomography,

*USD* US dollar

**Supplemental Table 3:** Manifestations associated with APDS costs inputs^1-43^

| Manifestation | Prevalence, mean (range), % | No. of episodes, mean (range) | Annual unit cost, $ | Annual complication cost, mean (range), $ | Source and assumptions |
| --- | --- | --- | --- | --- | --- |
| **Infections** | | | | | |
| Respiratory tract infections | 85 (80‒90) | 3.00 (1‒5) | 66.64 | 169.92 (53.31, 299.86) | Fendrick et al, 2003; Manifestations survey |
| Pneumonia | 37 (15‒67) | 1.17 (1‒2) |  | 4921.14 (1068.70, 30,232.72) | Manifestations survey |
| Pneumonia, inpatient | 26 (13‒60) |  | 35699.66 |  | Sato et al, 2013; Manifestations survey |
| Pneumonia, outpatient | 74 (87‒40) |  | 2854.84 |  | Sato et al, 2013; Manifestations survey |
| Sinusitis | 47 (0‒90) | 1.33 (0‒3) |  | 4742.46 (0.00, 20,578.87) | Manifestations survey |
| Chronic rhinosinusitis | 47 (0‒90) | 1.33 (0‒3) | 7621.80 |  | Caulley et al, 2015; Manifestations survey |
| Otitis media, total | 33 (0‒75) | 2.00 (0‒3) | 0.00 | 199.23 (0, 1006.45) | Manifestations survey |
| Otitis media, inpatient | 3 (0-10) |  | 2007.84 |  | Tong et al, 2018; Manifestations survey |
| Otitis media, outpatient | 97 (100‒90) |  | 250.84 |  | Tong et al, 2018; Manifestations survey |
| Otitis media, ED | 1 (0‒5) |  | 415.46 |  | Tong et al, 2018; Manifestations survey |
| Otitis needing tympanostomy tubes | 13 (0‒30) |  | 992.48 | 129.02 (0, 297.74) | Sjorgren et al, 2016; Manifestations survey |
| Dental/oral tissue abscesses | 19 (0‒50) |  | 534.46 | 101.55 (0, 267.23) | CMS PFS; Manifestations survey |
| Cellulitis | 14 (0‒50) | 0.83 (0‒1) | 804.55 | 91.63 (0, 402.28) | St. John et al, 2018; Manifestations survey |
| Meningitis | 4 (0‒10) | 0.67 (0‒1) | 15,670.13 | 365.64 (0, 1567.01) | Balada-Llasat et al, 2019; Manifestations survey |
| Endocarditis, total | 1 (0‒5) |  |  | 203.38 (0, 1082.86) | Manifestations survey |
| Endocarditis inpatient | 66 (6‒100) |  | 21,657.10 |  | Adams et al, 2022; Manifestations survey |
| Endocarditis outpatient | 34 (94‒0) |  | 2712.53 |  | Adams et al, 2022; Manifestations survey |
| Severe or persistent herpesvirus infection | 20 (0‒50) | 1.33 (0‒2) | 348.14 | 70.79 (0, 174.07) | Di Xia et al, 2018; Manifestations survey |
| EBV | 10 (7‒13) | 1.33 (0‒2) |  | 4.70 (0, 9.17) | Manifestations survey |
| EBV treatment with generics |  |  | 10.95 |  | Red Book; Manifestations survey |
| EBV diagnostic test |  |  | 24.31 |  | CMS LFS; Manifestations survey |
| CMV | 6 (3‒9) | 1.33 (0‒2) |  | 265.81 (0.00‒590.09) | Manifestations survey |
| CMV treatment with IVIG |  |  | 7683.98 |  | El Haddad et al, 2020; Manifestations survey |
| CMV treatment with foscarnet |  |  | 3037.80 |  | El Haddad et al, 2020; Manifestations survey |
| CMV treatment with ganciclovir |  |  | 1053.77 |  | El Haddad et al, 2020; Manifestations survey |
| CMV treatment with valganciclovir |  |  | 983.19 |  | El Haddad et al, 2020; Manifestations survey |
| HSV | 9 (6‒12) | 1.33 (0‒2) | 10.95 | 1.28 (0.00‒2.57) | Red Book; Manifestations survey |
| VZV | 6 (3‒9) | 1.33 (0‒2) | 10.95 | 0.91 (0.00‒2.03) | Red Book; Manifestations survey |
| Tonsillitis | 7 (0‒20) | 0.83 (0‒1) | 66.64 | 3.89 (0.00‒13.33) | Fendrick et al, 2003; Manifestations survey |
| Tonsillitis with tonsillectomy | 10 (0‒30) |  | 6646.07 | 653.53 (0.00‒1993.82) | HCUPnet, 2023; Manifestations survey |
| Ocular infections | 3 (0‒10) | 0.83 (0‒2) | 792.16 | 22.44 (0.00‒79.22) | Pepose et al, 2020; Manifestations survey |
| Septicemia | 6 (0‒20) |  | 23,546.48 | 1412.79 (0.00‒4709.30) | Torio et al, 2013; Manifestation survey |
| Fungal Infection, total | 6 (0‒10) | 1.50 (0‒5) |  | 2413.89 (0.00‒35,373.90) | Manifestations survey |
| Fungal infection inpatient | 36 (10‒100) |  | 70,747.81 |  | Benedict et al, 2019; Manifestation survey |
| Fungal infection outpatient | 64 (90‒0) |  | 346.56 |  | Benedict et al, 2019; Manifestation survey |
| Granulomatous lesions, total | 11 (0‒50) |  |  | 34.11 (0.00‒162.45) | Manifestations survey |
| CT scan |  |  | 313.95 |  | CMS PFS; Manifestations survey |
| Treatment cost with generics |  |  | 10.95 |  | Red Book; Manifestations survey |
| BCGosis | 1 (0‒5) |  | 39,082.25 | 455.96 (0.00‒1954.11) | HCUPnet, 2023; Manifestations survey |
| Lymphadenitis | 22 (0‒50) |  |  | 7.55 (0.00‒17.30) | Manifestations survey |
| Treatment with antibiotic (azithromycin) | 100 (100‒100) |  | 34.59 |  | Red Book; Manifestations survey |
| Minor infections | 68 (40‒100) |  | 1840.32 | 1257.56 (736.13‒1840.32) | Menzin et al, 2014; Manifestations survey |
| Major infections | 29 (10‒50) |  | 48,622.38 | 14,181.53 (4862.24‒ 24,311.19) | Menzin et al, 2014; Manifestations survey |
| **Hematologic** | | | | | |
| Splenomegaly | 67 (25‒100) |  |  | 3090.83 (78.49‒16,480.20) | Manifestations survey |
| Splenomegaly CT scan | 100 (100‒100) |  | 313.95 |  | CMS PFS; Manifestations survey |
| Splenomegaly nonsurgery treatment ‒ watchful waiting | 65 (100‒0) |  | 0.00 |  | Assumption; Manifestation survey |
| Splenomegaly treatment with steroids | 23 (0‒ 50) |  | 131.49 |  | Red Book; Manifestations survey |
| Splenomegaly nonsurgery treatment ‒Jakafi | 5 (0‒20) |  | 17,020.00 |  | Red Book; Manifestations survey |
| Splenomegaly nonsurgery treatment ‒ Rituxan | 8 (0‒30) |  | 42,321.67 |  | Schmier et al, 2017; Manifestation survey |
| Lymphadenopathy (diagnostic lymph node biopsy) | 71 (50‒100) |  | 637.43 | 451.51 (318.72‒637.43) | CMS PFS; Manifestations survey |
| Hepatomegaly | 34 (20‒50) |  |  | 1069.71 (62.79‒6538.10) | Manifestations survey |
| Hepatomegaly diagnostic | 100 (100‒100) |  | 313.95 |  | CMS PFS; Manifestations survey |
| Hepatomegaly treatment ‒ watchful waiting | 62 (100‒20) |  | 0.00 |  | Assumption; Manifestation survey |
| Hepatomegaly treatment ‒ steroid (generic) | 18 (0‒50) |  | 131.49 |  | Red Book; Manifestations survey |
| Hepatomegaly treatment ‒ Rituxan | 7 (0‒30) |  | 42,321.67 |  | Schmier et al, 2017; Manifestation survey |
| Hemolytic anemia treatment with steroids | 13 (0‒25) |  | 131.49 | 17.53 (0.00‒32.87) | Red Book; Manifestations survey |
| Nodular lymphoid hyperplasia | 17 (0‒50) |  |  | 73.42 (0.00‒220.26) | Manifestations survey |
| Nodular lymphoid hyperplasia-small bowel endoscopy |  |  | 208.85 |  | CMS PFS; Manifestations survey |
| Nodular lymphoid hyperplasia ‒ barium enema |  |  | 231.67 |  | CMS PFS; Manifestations survey |
| Pancytopenia | 9 (0‒30) |  |  | 1988.18 (0.00‒18,670.45) | Manifestations survey |
| Pancytopenia treatment with epoetin alfa | 18 (0‒50) |  | 1232.23 |  | Vekeman et al, 2007; Manifestations survey |
| Pancytopenia treatment with filgrastim | 17 (0‒50) |  | 16.00 |  | Lyman et al, 2009; Manifestation survey |
| Pancytopenia treatment with RBC transfusion | 28 (0‒80) |  | 66,407.89 |  | Betibeglogene autotemcel for β-thalassemia, 2022; Manifestations survey |
| Pancytopenia treatment with platelet transfusion | 26 (0‒80) |  | 10,605.52 |  | Betibeglogene autotemcel for β-thalassemia 2022; Manifestations survey |
| Immune thrombocytopenia | 14 (0‒30) |  |  | 8890.11 (0.00‒48,987.93) | Manifestations survey |
| Immune thrombocytopenia ‒ no treatment | 28 (1‒100) |  | 0.00 |  | Manifestations survey |
| Immune thrombocytopenia treatment with steroids | 50 (0‒80) |  | 131.49 |  | Red Book; Manifestations survey |
| Immune thrombocytopenia treatment with romiplostim (Nplate) | 20 (0‒50) |  | 197,016.06 |  | Patwardhan et al, 2021; Manifestation survey |
| Immune thrombocytopenia treatment with eltrombopag (Promacta) | 18 (0‒50) |  | 129,359.72 |  | Patwardhan et al, 2021; Manifestation survey |
| Factor XI deficiency | 3 (0‒10) |  | 522.57 | 14.63 (0.00‒52.26) | Klifto et al, 2024; Manifestation survey |
| Factor IX deficiency | 1 (0‒2) |  | 590,586.50 | 3937.24 (0.00‒11,811.73) | Hemophilia A and B, 2022 |
| Spherocytosis | 2 (0‒5) |  |  | 137.91 (0.00‒1076.64) | Manifestations survey |
| Spherocytosis treatment with epoetin alfa | 5 (0‒10) |  | 1232.23 |  | Vekeman et al, 2007; Manifestation survey |
| Spherocytosis treatment with transfusions pediatric | 5 (0‒12) |  | 44,472.71 |  | Betibeglogene autotemcel for β-thalassemia, 2022; Manifestations survey |
| Spherocytosis treatment with transfusions adults | 8 (0‒18) |  | 88,343.07 |  | Betibeglogene autotemcel for β-halassemia, 2022; Manifestations survey |
| Thrombotic thrombocytopenic purpura | 2 (0‒5) |  |  | 350.94 (0.00‒2983.72) | Manifestations survey |
| Thrombotic thrombocytopenic purpura treatment with plasma exchange | 22 (0‒80) |  | 4085.07 |  | Heatwole et al, 2011; Manifestation survey |
| Thrombotic thrombocytopenic purpura treatment with Cablivi | 7 (0‒20) |  | 282,032.00 |  | Goshua et al, 2021; Manifestatin survey |
| Cytopenia (cytopenia with steroids) | 31 (0‒50) |  | 131.49 | 40.54 (0.00‒65.75) | Red Book; Manifestations survey |
| Splenectomy | 2 (0‒5) |  | 19,463.40 | 389.27 (0.00‒ 973.17) | HCUPnet, 2023; Manifestations survey |
| **Malignancies** | | | | | |
| Hodgkin lymphoma | 3 (0‒5) |  | 53,039.33 | 1325.98 (0.00‒2651.97) | Huntington et al, 2018; Manifestation survey |
| Diffuse large B-cell lymphoma | 3 (0‒ 5) |  | 98,611.64 | 2958.35 (0.00‒4930.58) | Morrison et al, 2018; Manifestation survey |
| MALT lymphoma | 1 (0‒2) |  | 98,611.64 | 986.12 (0.00‒1972.23) | Morrison et al, 2018; Manifestation survey |
| Marginal zone B-cell lymphoma | 1 (0‒2) |  | 98,611.64 | 821.76 (0.00‒1972.23) | Morrison et al, 2018; Manifestation survey |
| Multiple lymphoma | 2 (0‒5) |  | 98,611.64 | 1479.17 (0.00‒4930.58) | Morrison et al, 2018; Manifestation survey |
| Other nonlymphoma malignancy ‒ acute myeloid leukemia | 0 (0‒0) |  | 98,537.44 | 0 (0‒0) | Huggar et al, 2022; Manifestation survey |
| **CNS (eg, neuropsychiatric, developmental delays)** | | | | | |
| Failure to thrive | 10 (0‒20) |  |  | 533.77 (0.00‒3099.97) | - |
| Failure to thrive treatment with gastrostomy | 17 (0‒50) |  | 18,046.42 |  | Yu et al, 2020; Manifestation survey |
| Failure to thrive treatment with gastrojejunostomy tube | 7 (0‒20) |  | 32,383.31 |  | Yu et al, 2020; Manifestation survey |
| Seizures | 5 (0‒10) |  | 6475.23 | 323.76 (0.00‒647.52) | Choi et al, 2019; Manifestation survey |
| Dysmorphic features | 3 (0‒10) |  | 966.05 | 25.76 (0.00‒96.61) | Dickerson et al, 2018; Manifestation survey |
| Brain biopsy | 1 (0‒2) |  | 1907.17 | 15.89 (0.00‒38.14) | CMS PFS; Manifestations survey |
| **Pulmonary** | | | | | |
| Bronchiectasis | 40 (10‒90) |  | 14,603.88 | 5792.87 (1460.39‒ 13,143.49) | HCUPnet, 2023; Manifestations survey |
| Interstitial lung disease | 13 (3‒25) |  | 23,320.88 | 3031.71 (699.63‒5830.22) | Mortimer et al, 2019; Manifestations survey |
| Atelectasis | 16 (0‒50) |  |  | 438.05 (0.00‒2252.69) | - |
| Atelectasis CT scan | 100 (100‒100) |  | 313.95 |  | CMS PFS; Manifestations survey |
| Atelectasis bronchoscopy | 100 (100‒100) |  | 353.51 |  | CMS PFS; Manifestations survey |
| Atelectasis ultrasound of the thorax | 100 (100‒100) |  | 77.20 |  | CMS PFS; Manifestations survey |
| Atelectasis treatment-continuous positive airway pressure | 11 (0‒20) |  | 18,803.55 |  | HCUPnet, 2023; Manifestations survey |
| Chest/thorax surgery | 3 (0‒5) |  | 26,220.35 | 655.51 (0.00‒1311.02) | Lacin et al, 2013; Manifestation survey |
| Lung resection | 2 (0‒5) |  | 26,220.35 | 393.31 (0.00‒1311.02) | Lacin et al, 2013; Manifestation survey |
| **Gastrointestinal** | | | | | |
| Enteropathy | 24 (20‒30) |  |  | 10,589.06 (202.21‒26,329.41) | Manifestations survey |
| Enteropathy ‒ colonoscopy | 100 (100‒100) |  | 1011.07 |  | CMS PFS; Manifestations survey |
| Enteropathy ‒ IBD treatment assumed | 21 (0‒50) |  | 24,307.58 |  | Park et al, 2020; Manifestations survey |
| Enteropathy ‒ steroids | 26 (0‒50) |  | 149,199.68 |  | Ricci et al, 2020; Manifestations survey |
| Diarrhea | 22 (0‒50) |  | 0.00 | 0 (0‒0) | Manifestation survey |
| Nodular regenerative hyperplasia of the liver | 6 (0‒11) |  | 552.62 | 33.16 (0.00‒60.79) | CMS PFS; Manifestations survey |
| Inflammatory bowel disease | 8 (0‒20) |  | 24,307.58 | 2025.63 (0.00‒4861.52) | Park et al, 2020; Manifestations survey |
| Pancreatic insufficiency | 4 (0‒5) |  |  | 9.76 (0.00‒32.32) | Manifestations survey |
| Pancreatic insufficiency ‒ fecal test | 100 (100‒100) |  | 30.78 |  | CMS LFS; Manifestations survey |
| Pancreatic insufficiency ‒ pancreatic enzyme treatment | 31 (0‒80) |  | 769.62 |  | Gardner et al, 2014; Manifestations survey |
| EoE/EGID | 4 (0‒10) |  |  | 2646.61 (0.00‒13,865.93) | Manifestations survey |
| EoE/EGID ‒ endoscopy | 100 (100‒100) |  | 208.85 |  | CMS PFS;  Manifestation survey |
| EoE/EGID ‒ Flovent (fluticasone) treatment | 100 (100‒100) |  | 13,033.06 |  | HCUPnet, 2023; Manifestations survey |
| GI surgery | 6 (0‒20) |  | 19,059.46 | 1080.04 (0.00‒3811.89) | HCUPnet, 2023; Manifestations survey |
| Liver biopsy | 7 (0‒20) |  | 552.62 | 38.68 (0.00‒110.52) | CMS PFS; Manifestations survey |
| Solid organ transplant | 2 (0‒5) |  | 937,903.48 | 15,631.72 (0.00‒46,895.17) | Bentley et al, 2017; Manifestations survey |
| GVHD, after solid organ transplant | 21 (0‒50) |  | 149,199.68 | 518.05 (0.00‒3729.99) | Ricci et al, 2020; Manifestation survey |
| **Dermatology** | | | | | |
| Dermatitis | 18 (0‒50) |  |  | 1035.93 (0.00‒5481.71) | Manifestation survey |
| Eczema or dermatitis ‒ mild treatment with generic or OTC medications | 71 (15‒100) |  | 7.20 |  | Red Book; Manifestations survey |
| Eczema or dermatitis‒moderate or severe treatment | 26 (5‒50) |  | 21,912.45 |  | Eichenfield et al, 2020; Manifestations survey |
| **Endocrine** | | | | | |
| Adrenal insufficiency | 4 (0‒10) |  |  | 2.12 (0.00‒5.09) | Manifestations survey |
| Adrenal insufficiency ‒ ACTH stimulation test | 100 (100‒100) |  | 43.71 |  | CMS LFS; Manifestations survey |
| Adrenal insufficiency ‒ treatment with generic glucocorticoids | 100 (100‒100) |  | 7.20 |  | Red Book; Manifestations survey |
| Diabetes | 10 (2‒25) |  |  | 1134.19 (223.12‒2788.99) | Manifestations survey |
| Diabetes treatment | 100 (100‒100) |  | 11,082.84 |  | American Diabetes Association, 2018; Manifestations survey |
| Diabetes A1C test | 100 (100‒100) | 4 (4-4) | 13.01 |  | CMS LFS; Manifestations survey |
| Diabetes blood sugar test | 100 (100‒100) | 4 (4-4) | 5.27 |  | CMS LFS; Manifestations survey |
| Hypothyroidism | 11 (0‒25) |  |  | 1446.88 (0.00‒3444.95) | Manifestations survey |
| Hypothyroidism treatment with generics, eg, levothyroxine | 100 (100‒100) |  | 13,689.73 |  | HCUPnet, 2023; Manifestations survey |
| Hypothyroidism TSH test | 100 (100‒100) | 4 (4-4) | 22.51 |  | CMS LFS; Manifestations survey |
| **Other (eg, arthritis, allergy, asthma, cardiac)** | | | | | |
| Cardiomyopathy | 6 (0‒25) |  |  | 1256.85 (0.00‒5712.98) | Manifestations survey |
| Cardiomyopathy x-ray |  |  | 50.52 |  | CMS PFS; Manifestations survey |
| Cardiomyopathy echocardiogram |  |  | 262.08 |  | CMS PFS; Manifestations survey |
| Cardiomyopathy electrocardiogram |  |  | 21.33 |  | CMS PFS; Manifestations survey |
| Cardiomyopathy treatment |  |  | 22,517.98 |  | Butzner et al, 2022; Manifestations survey |
| Dysgerminoma | 1 (0‒2) |  |  | 231.45 (0.00‒462.89) | Manifestations survey |
| Dysgerminoma CT scan |  |  | 313.95 |  | CMS PFS; Manifestations survey |
| Dysgerminoma MRI |  |  | 278.36 |  | CMS PFS; Manifestations survey |
| Dysgerminoma treatment ‒ ovary removal (salpingo-oophorectomy) |  |  | 22,552.21 |  | HCUPnet, 2023; Manifestations survey |
| Ehlers-Danlos | 2 (0‒5) |  |  | 678.70 (0.00‒2262.34) | Manifestations survey |
| Ehlers-Danlos biopsy |  |  | 421.94 |  | CMS PFS; Manifestations survey |
| Ehlers-Danlos x-ray |  |  | 50.52 |  | CMS PFS; Manifestations survey |
| Ehlers-Danlos CT scan |  |  | 313.95 |  | CMS PFS; Manifestations survey |
| Ehlers-Danlos treatment |  |  | 44,460.43 |  | HCUPnet, 2023; Manifestations survey |
| Asthma | 37 (5‒100) |  | 8907.86 | 3266.22 (445.39‒8907.86) | Nurmagambetov et al, 2018; Manifestation survey |
| Allergy | 27 (5‒60) |  | 9992.89 | 2681.43 (499.64‒5995.73) | HCUPnet 2023;  Manifestations survey |
| Arthritis | 11 (0‒30) |  |  | 1136.28 (0.00‒3007.80) | Manifestation survey |
| Arthritis DMARDs | 80 (80‒80) |  | 629.12 |  | Schmier et al, 2017; Manifestation survey |
| Arthritis biologics | 20 (20‒20) |  | 47,613.46 |  | Schmier et al, 2017; Manifestation survey |

*ACTH* adrenocorticotropic hormone, *APDS* activated phosphoinositide 3-kinase delta syndrome, *BCGosis* bacille Calmette-Guérin, *CMS* Centers for Medicare & Medicaid Services, *CMV* cytomegalovirus, *CNS* central nervous system, *CT* computed tomography, *DMARDs* disease-modifying antirheumatic drugs, *EBV* Epstein-Barr virus, *ED* emergency department, *EGID* eosinophilic gastrointestinal disease, *EoE* eosinophilic esophagitis, *GI* gastrointestinal, *GVHD* graft-vs-host disease, *GVHD* graft vs host disease, *HCUP* Healthcare Cost and Utilization Project, *HSV* herpes simplex virus, *IBD* inflammatory bowel disease, *IVIG* intravenous immunoglobulin, *LFS* laboratory fee schedule; *MALT* mucosa-associated lymphoid tissue, *MRI* magnetic resonance imaging, *OTC* over-the-counter, *PFS* physician fee schedule; *RBC* red blood cell, *TSH* thyroid stimulating hormone, *VZV* varicella-zoster virus

**Supplemental Fig 1:** Methodology for Calculating Manifestation Costs in Patients With APDS. The estimated costs per patient have accounted for founding in the calculations. *APDS* activated phosphoinositide 3-kinase delta syndrome, *USD* US dollar. ^a^Bolded numbers represent the low and high range.~~
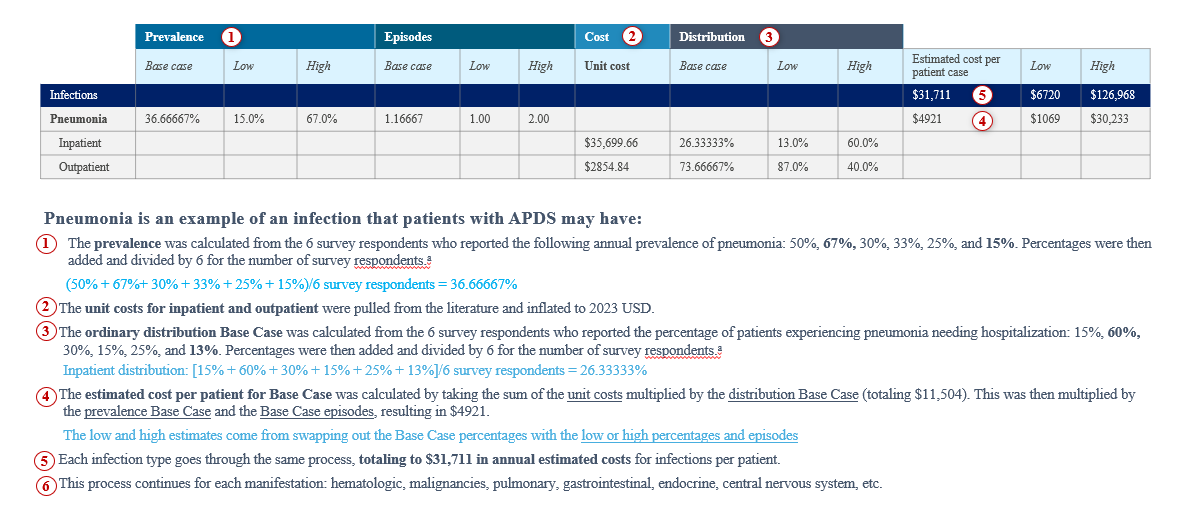
~~

**Supplementary References**

1. Centers for Medicare & Medicaid Services. Laboratory Fee Schedule. Baltimore, MD: CMS; 2023. Accessed February 16, 2024. https://www.cms.gov/medicare/payment/fee-schedules/clinical-laboratory-fee-schedule-clfs

2. Centers for Medicare & Medicaid Services. Physician Fee Schedule. Baltimore, MD: CMS; 2023. Accessed February 16, 2024. https://www.cms.gov/medicare/physician-fee-schedule/search

3. Betibeglogene Autotemcel for Beta Thalassemia: Final Policy Recommendations. Institute for Clinical and Economic Review. July 19, 2022. https://icer.org/wp-content/uploads/2022/07/ICER_BetaThalassemia_PolicyRecommendations_07192022.pdf.

4. IBM Micromedex RED BOOK. IBM Corporation. Greenwood Village, CO, USA. Accessed February 16, 2024.

5. HCUPnet, Healthcare Cost and Utlization Project. Agency for Healthcare Research and Quality, Rockville, MD. Accessed December 14, 2023. https://datatools.ahrq.gov/hcupnet/.

6. Hemophilia A and B. Institute for Clinical and Economic Review. Public Meeting – November 2022. Meeting Materials available at: https://icer.org/wp-content/uploads/2022/05/ICER_Hemophilia_Final_Report_12222022.pdf.

7. Adams JW, Savinkina A, Hudspeth JC, et al. Simulated cost-effectiveness and long-term clinical outcomes of addiction care and antibiotic therapy strategies for patients with injection drug use-associated infective endocarditis. *JAMA Netw Open*. 2022;5(2):e220541.

8. Balada-Llasat JM, Rosenthal N, Hasbun R, et al. Cost of managing meningitis and encephalitis among infants and children in the United States. *Diagn Microbiol Infect Dis*. Apr 2019;93(4):349-354.

9. Benedict K, Jackson BR, Chiller T, Beer KD. Estimation of direct healthcare costs of fungal diseases in the United States. *Clin Infect Dis*. May 17 2019;68(11):1791-1797.

10. Bentley TS, Phillips SJ. Milliman Research Report. 2017 U.S. organ and tissue transplant cost estimates and discussion. Accessed February 16, 2024. https://www.milliman.com/en/insight/2017-us-organ-and-tissue-transplant-cost-estimates-and-discussion.

11. Butzner M, Maron M, Sarocco P, et al. Healthcare resource utilization and cost of obstructive hypertrophic cardiomyopathy in a US population. *Am Heart J Plus*. Jan 2022;13:100089.

12. Caulley L, Thavorn K, Rudmik L, Cameron C, Kilty SJ. Direct costs of adult chronic rhinosinusitis by using 4 methods of estimation: Results of the US Medical Expenditure Panel Survey. *J Allergy Clin Immunol*. Dec 2015;136(6):1517-1522.

13. Choi H, Mohit B. Cost-effectiveness of screening for HLA-B*1502 prior to initiation of carbamazepine in epilepsy patients of Asian ancestry in the United States. *Epilepsia*. Jul 2019;60(7):1472-1481.

14. El Haddad L, Ghantoji SS, Park AK, et al. Clinical and economic burden of pre-emptive therapy of cytomegalovirus infection in hospitalized allogeneic hematopoietic cell transplant recipients. *J Med Virol*. Jan 2020;92(1):86-95.

15. Goshua G, Sinha P, Hendrickson JE, Tormey C, Bendapudi PK, Lee AI. Cost effectiveness of caplacizumab in acquired thrombotic thrombocytopenic purpura. *Blood*. Feb 18 2021;137(7):969-976.

16. Di Xia F, Fuhlbrigge M, Dommasch E, Joyce C, Mostaghimi A. Cost of routine herpes simplex virus infection visits to U.S. Emergency Departments 2006-2013. *West J Emerg Med*. Jul 2018;19(4):689-692.

17. Dickerson JF, Lynch FL, Leo MC, DeBar LL, Pearson J, Clarke GN. Cost-effectiveness of cognitive behavioral therapy for depressed youth declining antidepressants. *Pediatrics*. Feb 2018;141(2)

18. Eichenfield LF, DiBonaventura M, Xenakis J, et al. Costs and treatment patterns among patients with atopic dermatitis using advanced therapies in the United States: analysis of a retrospective claims database. *Dermatol Ther (Heidelb)*. Aug 2020;10(4):791-806.

19. Fendrick AM, Monto AS, Nightengale B, Sarnes M. The economic burden of non-influenza-related viral respiratory tract infection in the United States. *Arch Intern Med*. Feb 24 2003;163(4):487-494.

20. Gardner TB, Munson JC, Morden NE. The FDA and prescription pancreatic enzyme product cost. *Am J Gastroenterol*. May 2014;109(5):624-625.

21. Heatwole C, Johnson N, Holloway R, Noyes K. Plasma exchange versus intravenous immunoglobulin for myasthenia gravis crisis: an acute hospital cost comparison study. *J Clin Neuromuscul Dis*. Dec 2011;13(2):85-94.

22. Huggar D, Knoth RL, Copher R, et al. Economic burden in US patients with newly diagnosed acute myeloid leukemia receiving intensive induction chemotherapy. *Future Oncol*. Oct 2022;18(32):3609-3621.

23. Huntington SF, von Keudell G, Davidoff AJ, Gross CP, Prasad SA. Cost-effectiveness analysis of brentuximab vedotin with chemotherapy in newly diagnosed stage III and IV Hodgkin lymphoma. *J Clin Oncol*. Oct 4 2018;36(33):Jco1800122.

24. Klifto KM, Klifto CS, Pidgeon TS, Richard MJ, Ruch DS, Colbert SH. Platelet-rich plasma versus corticosteroid injections for the treatment of mild-to-moderate carpal tunnel syndrome: a Markov cost-effectiveness decision analysis. *Hand (N Y)*. Jan 2024;19(1):113-127.

25. Lacin T, Swanson S. Current costs of video-assisted thoracic surgery (VATS) lobectomy. *J Thorac Dis*. Aug 2013;5 Suppl 3(Suppl 3):S190-193.

26. Lyman GH, Lalla A, Barron RL, Dubois RW. Cost-effectiveness of pegfilgrastim versus filgrastim primary prophylaxis in women with early-stage breast cancer receiving chemotherapy in the United States. *Clin Ther*. May 2009;31(5):1092-1104.

27. Mortimer K, Hartmann N, Chan C, Norman H, Wallace L, Enger C. Characterizing idiopathic pulmonary fibrosis patients using US Medicare-advantage health plan claims data. *BMC Pulm Med*. Jan 10 2019;19(1):11.

28. Park KT, Ehrlich OG, Allen JI, et al. The cost of inflammatory bowel disease: an initiative from the Crohn's & Colitis Foundation. *Inflamm Bowel Dis*. Jan 1 2020;26(1):1-10.

29. Patwardhan P, Proudman D, Allen J, Lucas S, Nellesen D. Cost-minimization analysis comparing eltrombopag vs romiplostim for adults with chronic immune thrombocytopenia. *J Manag Care Spec Pharm*. Oct 2021;27(10):1447-1456.

30. Pepose JS, Sarda SP, Cheng WY, et al. Direct and indirect costs of infectious conjunctivitis in a commercially insured population in the United States. *Clin Ophthalmol*. 2020;14:377-387.

31. Nurmagambetov T, Kuwahara R, Garbe P. The economic burden of asthma in the United States, 2008-2013. *Ann Am Thorac Soc*. Mar 2018;15(3):348-356.

32. Morrison VA, Bell JA, Hamilton L, et al. Economic burden of patients with diffuse large B-cell and follicular lymphoma treated in the USA. *Future Oncol*. Oct 2018;14(25):2627-2642.

33. Menzin J, Sussman M, Munsell M, Zbrozek A. Economic impact of infections among patients with primary immunodeficiency disease receiving IVIG therapy. *Clinicoecon Outcomes Res*. 2014;6:297-302.

34. Ricci A, Jin Z, Broglie L, et al. Healthcare utilization and financial impact of acute-graft-versus host disease among children undergoing allogeneic hematopoietic cell transplantation. *Bone Marrow Transplant*. Feb 2020;55(2):384-392.

35. Sato R, Gomez Rey G, Nelson S, Pinsky B. Community-acquired pneumonia episode costs by age and risk in commercially insured US adults aged ≥50 years. *Appl Health Econ Health Policy*. Jun 2013;11(3):251-258.

36. Schmier J, Ogden K, Nickman N, et al. Costs of providing infusion therapy for rheumatoid arthritis in a hospital-based infusion center setting. *Clin Ther*. Aug 2017;39(8):1600-1617.

37. Sjogren PP, Gale C, Henrichsen J, et al. Variation in costs among surgeons and hospitals in Pediatric tympanostomy tube placement. *Laryngoscope*. Aug 2016;126(8):1935-1939.

38. St John J, Strazzula L, Vedak P, Kroshinsky D. Estimating the health care costs associated with recurrent cellulitis managed in the outpatient setting. *J Am Acad Dermatol*. Apr 2018;78(4):749-753.

39. Tong S, Amand C, Kieffer A, Kyaw MH. Trends in healthcare utilization and costs associated with acute otitis media in the United States during 2008-2014. *BMC Health Serv Res*. May 2 2018;18(1):318.

40. Torio CM, Moore BJ. National Inpatient Hospital Costs: The Most Expensive Conditions by Payer, 2013. *Healthcare Cost and Utilization Project (HCUP) Statistical Briefs*. Agency for Healthcare Research and Quality (US); 2006.

41. Vekeman F, McKenzie RS, Lefebvre P, et al. Dose and cost comparison of erythropoietic agents in the inpatient hospital setting. *Am J Health Syst Pharm*. Sep 15 2007;64(18):1943-1949.

42. Yu YR, Cunningham ME, DeMello AS, et al. Cost-effectiveness analysis of the surgical management of infants less than one year of age with feeding difficulties. *J Pediatr Surg*. Jan 2020;55(1):187-193.

43. American Diabetes Association. Economic costs of diabetes in the U.S. in 2017. *Diabetes Care*. 2018;41(5):917-928.
